# HIV/AIDS related commodities supply chain management in public health facilities of Addis Ababa, Ethiopia: a cross-sectional survey

**DOI:** 10.1186/s40545-016-0060-z

**Published:** 2016-03-31

**Authors:** Eyerusalem Berhanemeskel, Gebremedhin Beedemariam, Teferi Gedif Fenta

**Affiliations:** Departement of Pharmaceutics and Social Pharmacy, School of Pharmacy, College of Health Sciences, Addis Ababa University, Ethiopia, P. O. Box: 1176, Addis Ababa, Ethiopia

**Keywords:** HIV/AIDS, ARV medicines, HIV test kits, Supply chain management, Pharmaceutical logistic, Ethiopia

## Abstract

**Background:**

A wide range of pharmaceutical products are needed for diagnosis, treatment, and prevention of HIV/AIDS. However, interrupted supplies and stock-outs are the major challenges in the supply chain of ARV medicines and related commodities. The aim of this study was to assess the supply chain management of HIV/AIDS related commodities in public health facilities of Addis Ababa, Ethiopia.

**Methods:**

A descriptive cross-sectional survey complemented by qualitative method was conducted in 24 public health facilities (4 hospitals and 20 health centers). A semi-structured questionnaire and observation check list were used to collect data on HIV/AIDS related service, reporting and ordering; receiving, transportation and storage condition of ARV medicines and test kits; and supportive supervision and logistics management information system. In addition, in-depth interview with flexible probing techniques was used to complement the quantitative data with emphasis to the storage condition of ARV medicines and test kits. Quantitative data was analyzed using SPSS version-20. Analysis of qualitative data involved rigorous reading of transcripts in order to identify key themes and data was analyzed using thematic approach.

**Results:**

The study revealed that 16 health centers and one hospital had recorded and reported patient medication record. Six months prior to the study, 14 health centers and 2 hospitals had stopped VCT services for one time or more. Three hospitals and 18 health centers claimed to have been able to submit the requisition and report concerning ARV medicines to Pharmaceutical Fund and Supply Agency according to the specific reporting period. More than three-fourth of the health centers had one or more emergency order of ARV medicines on the day of visit, while all of hospitals had emergency order more than 3 times within 6 months prior to the study. All of the hospitals and nearly half of the health centers had an emergency order of test kits more than 3 times in the past 6 months. Overall, nearly 3/4th of the health facilities faced stock-out of one or more ARV medicines and test kits on the day of visit.

**Conclusion:**

There was no adequate data on patient medication record and stock status of HIV/AIDS related commodities. Moreover there were frequent stock-outs of ARV medicines and HIV test kits, which was an indicator of the weak supply chain management. Hospitals and health centers, therefore, should devise a system to capture and make use of patient medication record and stock status information so as to ensure continuous supply of the commodities.

## Background

The human immune virus (HIV) epidemic remains a major global public health challenge including in Ethiopia. According to the 2014 estimate, Ethiopia had 793,700 people living with HIV with 15, 700 new HIV infections and 35, 600 AIDS-related deaths. The national HIV prevalence was 1.14 % in 2014 and it is declining significantly varying by age, gender and geographical location [1].

In the early 1980s when the AIDS epidemic began, people living with HIV were not likely to live more than a few years [2]. However, with the development of safe and effective medicines, HIV positive people now have longer and healthier lives [2, 3]. Initially, resource limited countries could not afford to provide antiretroviral therapy (ART) for their populations, and the life expectancy of HIV positive people remained low [4]. However, efforts have been made to make it more affordable within low- and middle-income countries [3, 5].

Supply chain management of essential health commodities, including high-value medicines like Antiretroviral (ARV) medicines, involves a series of activities to guarantee the continuous flow of products from the manufacture to consumers [6]. The nature of ART and the specific characteristics of ARV medicines and how they are used pose particular challenges for managing the supply chain for ARV medicines [7].

Effective pharmaceutical supply management and inventory control avoid stock out, loss due to unnecessary expiry, theft and ensure that the desired pharmaceutical products are available at all times in adequate quantity [8]. But in many low and middle income countries (LMICs), the capacity of the pharmaceutical supply management system has always been challenging and weak. The ARV supply chain management has become increasingly difficult due to increasing number of people on ART, increasing number of sites providing ART and a greater diversity of different ARV regimen [9].

Moreover, there are certain common challenges associated with the quantification of ARV medicines and supplies mainly in LMICs. Data on ART services and ARV medicine supply are limited and when available, are often unreliable or insufficient to be used for quantifying ARV medicine requirements [7]. An accurate quantification based on reliable data is essential for all health commodities but more so for HIV/AIDS related commodities because uninterrupted access for patients must be ensured [9]. A pilot study done in Ethiopia, however, showed that out of the 48 hospitals and health centers, 10(21 %) of the institutions didn’t have HIV medicines and out of 27 health posts, 9 (33 %) did not have rapid diagnostic tests [10]. This shortage of critical medicines and supplies in health facilities may compromise appropriate clinical management which ultimately increases mortality and development of resistance pathogens causing a detrimental public health impact [11].

One of the major reasons that medicines are wasted is that they may have expired without anyone noticing that the shelf life date was approaching. This type of lose, however, is not acceptable to pharmaceuticals such as ARV medicines, which are very expensive [8]. Besides, due to poor handling of the available medicines and other pharmaceutical products by the patients and professionals, there is also a great loss of resources.

In addition to this, the 2010 World Health Organization (WHO) guideline and the latest 2010 Ethiopian standard treatment guideline recommend that ART should be initiated when the CD4 count falls below 350/μl for WHO stage 3 disease and should be initiated irrespective of CD4 count for stage 4 disease. If CD4 count is not available it should be initiated irrespective of total lymphocyte count [4, 12]. So it may lead to a drastic increase of the number of patients who are eligible for ART and thus creating an enormous burden to national health care system and health facilities [13, 14].

Assessing the supply chain of ARV medicines and HIV test kits is indispensible to improve access and thus provide quality services. Little has been done in this regard. However a pilot study done by Daniel et al. (10) tried to assess the availability of HIV medicines but did not address the other components of the supply chain management. This study was therefore conducted with the aim of comprehensively assessing the supply chain management of HIV/AIDS related commodities in public health facilities of Addis Ababa, Ethiopia.

## Methods

A descriptive cross-sectional survey employing both quantitative and qualitative data collection technique was conducted in the selected public health facilities. All public health facilities providing voluntary counseling and testing (VCT), prevention of mother to child transmission (PMTCT) and ART services were the source facilities of the study. All healthcare professionals working in ART clinic of the selected health facilities and all documents that were used to manage the supply chain of HIV/AIDS related commodities were used as sources of information.

The numbers of health facilities to be included in the study were calculated by using the Logistic Indicators Assessment Tool (LIAT) for ARV medicines and Test kits [14, 15]. At the time of survey, a total of 11 public hospitals and 37 health centers were providing ART and VCT services. Of these, 20 health centers; two from each of the ten sub-cities where one health center with the highest number of patients on ART treatment and the other with the lowest patients burden were selected for the study. Selection of study hospitals was also done based on their ownership and patient burden i.e. hospitals were administratively stratified into those administered by the Regional Health Bureau and the Federal Ministry of Health (FMoH) and then selection of the health facilities was made by extreme sampling. Accordingly four governmental hospitals, two from FMoH and two from Regional Health Bureau, were included in the study. Two of the selected hospitals, one from each administrative category, had the lowest patient load while two of the remaining hospitals again one from each had the highest burden of patients.

A semi-structured questionnaire and observation check list were used to collect the quantitative data. A modified version of the LIAT for ARV medicines and test kits was used as a data collection tool [15, 16]. A total of 14 ARV medicines and five HIV test kits were selected for this assessment. A six month data (May 2013 to October 2013) were taken from bin card and VCT daily register to see the pattern of VCT service and stock status in hospitals and health centers. VCT daily register, ARV medicines and patient information sheets (PIS), ARV medicines dispensing register, patient tracking charts, ARV medicines dispensing register for post exposure prophylaxis, ARV medicines dispensing register for emergency supply, medicine reporting and requisition format (RRF), Model 19 (receiving voucher), bin card, medicine and supply expiry date tracking charts and temperature recording charts were the major documents checked and reviewed to get the required information.

An in-depth interview with flexible probing techniques was designed to collect the qualitative data from the key informants. The head of pharmacy departments, ART store managers, ART dispensers, Laboratory heads, VCT staff and ART coordinator from the selected hospitals and health centers were purposively identified as key informants for the study.

After the data was manually checked for completeness and consistencies, it was entered and analyzed by using SPSS version 20. Descriptive statistics including mean, percentage and standard deviations was used to present the quantitative data. The qualitative data analysis involved an intensive reading through the interview in order to identify key themes. Audio-recorded interviews were transcribed verbatim and the raw data was categorized under pre-developed coded themes and sub themes. A thematic analysis was then used to analyze the data.

Ethical approval was obtained from the Ethics Review Board of the School of Pharmacy, Addis Ababa University, Addis Ababa Regional Health Bureau and from the respective health facilities. Besides, a verbal consent was obtained from all participants before starting the actual data collection. Confidentiality and anonymity of information was maintained throughout the data collection and analysis period by not linking personal identifiers in the data presentations.

## Results

### HIV/AIDS related services

A total of 24 health facilities were visited during this assessment; of which 4 were hospitals and 20 were health centers. All selected facilities were providing VCT, PMTCT and ART services. The selected health centers had an experience on VCT service provision for an average of 9.1 years (SD = 1.4 and range from 6 to 11 years) and ART service provision for a mean period of 6.9 years (SD = 0.8 and range from 5 to 8 years); while hospitals had longer experience of providing VCT and ART services, 10.3 years (SD = 1.5 and range from 9 to 12 years) and 9 years (SD = 1.6 and range from 7 to 11 years) respectively.

A majority (65 %) of the health centers and half of the hospitals were not providing VCT service on the day of visit. Moreover, the VCT service was interrupted at least once in 14(70 %) of the health centers and 2(50 %) of the hospitals within six months prior to the commencement of the survey. When facilities had shortage of HIV test kits, they conducted tests only for emergency cases or for PMTCT. In the past six months prior to the study, VCT service was not provided by the health centers for longer period which averaging 39.8 ± 32.8 (range from 0 to 98) days compared to hospitals, 6.8 ± 11 (range from 0 to 23) days. Mostly the interruption was associated with stock-outs of HIV (1 + 2) Antibody Colloidal Gold (KHB) but shortage of stat pack and blood lancet were also mentioned as additional factors.

Regarding the ART services, majority (80 %) of the health centers and all of the hospitals had lists of recommended ARV medicine regimens to be prescribed and dispensed. A majority 16(80 %) of the health centers and only one hospital knew and reported their patient medication record. The rest, 4 health centers and 1 hospital were using ART clinic data to report to higher level. Two hospitals claimed that they were reporting the data from the ART pharmacy without retaining the copy of the reported data.

The key informants’ interview with the heads of pharmacy departments and the data clerks in two of the hospitals revealed that their Electronic Dispensing Tool (EDT) was not working appropriately and thus both the ART pharmacist and the data clerk were facing difficulties in using the patient database. In addition to this, they said that they wouldn’t use any paper based format to register patient data because of high patient burden. In another hospital, the head of the pharmacy and the ART pharmacist mentioned that they were only able to enter patient information without analyzing the data for further reporting. In addition to this, they also mentioned that due to negligence of the professionals and other factors, the data in EDT was not reliable. Even though they have been using PIS, they didn’t use it appropriately.

All of the health facilities had EDT to dispense medicines to the patient and all, except one, of them were applying the EDT in their daily work. A majority 19(95 %) of health centers and 3/4^th^ of the hospitals used both EDT and PIS to record the amount of medicines dispensed to the patients and other patient information. But, Dispensing Register was available and used only in 14 health centers and none of the hospitals. A majority of them had been dispensing a dose that ranges from 15 days to 3 months, depending on patients’ condition. Erratic supply of medicines in some health facilities, however, was reported by the ART pharmacists that forced them to dispense medicines for a week, 3 days and even for a day; especially for TDF/3TC based regimen. Sometimes, they mentioned that they even referred patients to other health facilities.

Eighteen health centers and 2 hospitals used separate register for post exposure prophylaxis and for emergency. Patient Tracking Chart was being used by only 10 health centers and 2 hospitals. Nevertheless, absentee patients tracked by this chart were not called to attend the health facility mainly due to lack of telephone in these health facilities.

### Reporting and ordering ARV medicines and HIV test kits

Store manager in one of the 20 health centers was not available during the study time and hence 19 key informants from the health centers and 4 from hospitals were considered. All health facilities reported using RRF to report consumption and order ARV medicines from the Pharmaceutical Fund and Supply Agency (PFSA) Regional Hub.

In a majority (89.5 %) of the health facilities, RRF was prepared and reported by the store manager alone. Thus, the store managers were responsible for determining the quantity of medicines. In the rest of the facilities, both the store manager and the head pharmacist claimed to have been involved in quantification. A majority (94.7 %) of the health centers and 3/4^th^ of hospitals’ store managers claimed that they had submitted their last report according to the schedule. A majority of the store managers 15(78.9 %) in health centers and 3(75 %) in hospitals had training on integrated pharmaceutical logistic system (IPLS).

A majority of the facilities had emergency orders in the past 6 months prior to the study. Only 3(15.8 %) of the health centers didn’t have emergency orders. On the other hand, all of the hospitals reported that they had emergency orders for more than 3 times within 6 months prior to the study (Table 1).

**Table 1:** Frequency of emergency orders for ARV drugs and HIV test kits in the health facilities, Addis Ababa, 2013

		Proportion by type of health facility	
		Health centers; n (%)	Hospitals; n (%)
Frequency of emergency orders encountered for ARV in the last 6 months			
	No emergency order	3(15.8)	0 (0.00)
	One emergency order	4(21.1)	0 (0.00)
	Two emergency orders	2(10.5)	0 (0.00)
	Three emergency orders	6(31.6)	0 (0.00)
	More than three emergency order	4(21.1)	4(100)
Frequency of emergency orders encountered for HIV test kits in the last 6 months			
	No emergency order	1(5.3)	0 (0.00)
	One emergency order	3(15.8)	0 (0.00)
	Two emergency orders	2(10.5)	0 (0.00)
	Three emergency orders	2(10.5)	0 (0.00)
	More than three emergency order	9(47.4)	4(100)
	Do not know	2(10.5)	0 (0.00)

The reporting and requisition of test kits was done in combination with the ARV medicines by the main store manager in all the studied health facilities except one hospital and two health centers. The hospital had a separate store for test kits, so the RRF was prepared by separate store manager. In the two health centers the RRF was prepared by the laboratory head that never had training for the purpose. All hospitals reported the use of standard method to determine the quantity of HIV test kits but only 9(47.3 %) of the health centers did use the same during the quantification process. Most health centers mentioned the practice of rough estimation since they were unable to calculate the exact quantity of test kits due to frequent supply interruption. Generally, the reporting and the requisition of HIV test kits were more organized in hospitals compared with health centers.

All health facilities, except one, had emergency order of test kits in the past six months prior to the study. All of the hospitals and nearly half (47.4 %) of the health centers had an emergency order for more than three times in the past six months. They all agreed that in the majority of the cases they received the test kits on emergency order (Table 1).

### Receiving and transportation of ARV medicines and test kits

All of the hospitals reported that they were not always getting the required quantity of ARV medicines and only 1 of the health center was always able to get the quantified amount (Table 2). Regarding the average time interval between ordering and receiving of the medicines, three-fourth of the hospitals and 13(68.4 %) of the health centers received the products ordered between two weeks to one month of their order point (Table 2). But review of the health facilities’ last report had showed that, on average the lead time was 37.5 ± 17.1 days and 34.2 ± 18 days in hospitals and health centers, respectively.

**Table 2:** Frequency of receiving the ordered quantity of ARV drugs and Average lead time for ARVs in hospitals and health centers, Addis Ababa, 2013

		Health centers; n (%)	Hospitals; n (%)
How frequently did you receive the amount of ARV drugs you ordered?			
	Always	1 (5.3)	0 (0.00)
	Most of the time	12 (63.2)	1 (25.0)
	Sometimes	6 (31.6)	3 (75.0)
	Never	0 (0.00)	0 (0.00)
Average lead time between ordering and receiving ARV drugs			
	Less than two weeks	3 (15.8)	1 (25.0)
	2 weeks to 1 month	13 (68.4)	3 (75.0)
	1 month to 2 months	2 (10.5)	0 (0.00)
	More than 2 months	1 (5.3)	0 (0.00)

During the last order time, the average number of ordered ARV product was found to be 6. The mean percentage difference between quantity ordered and received was high for 3TC300/TDF300 (69.6 % ±17 %, (55 %, 93 %)) and D4T12/3TC60 (69.4 % ± 33.3 %, (45.8 %, 93 %)) in hospitals while it was high for D4T6/3TC30/NVP 50 (110.9 % ± 193.8 %, (0 %, 400 %)) and 3TC300/TDF300 (51.7 % ± 33.8 %, (0 %, 99 %)) in health centers for last report period (Table 3).

**Table 3:** Stock-out status of ARVs within 6 months prior to the study and percentage difference between ordered and received quantities of ARVs in health centers and hospitals, Addis Ababa, 2013

ARV drugs	Stock-out days of ARVs within 6 months prior to the study		Percentage difference between ordered and received quantities of ARVS			
	Health centers	Hospitals	Health centers		Hospitals	
	Average number of days ± SD	Average number of days ± SD	Mean percentage difference ± SD	(Min, Max)	Mean percentage difference ± SD	(min, max)
EFV50	12 ± 21	0	0	0	^a^	^a^
EFV200	10.1 ± 28	0	9.5 ± 21.2	(0, 47.6)	0	0
EFV600	18.8 ± 25	0	47 ± 32.3	(0, 80.7)	2.9 ± 2.7	(0, 11.5)
3TC300/TDF300	45.9 ± 45.5	42.5 ± 60.1	51.7 ± 33.8	(0, 99)	69.6 ± 17	(55, 93.4)
NVP 200	16.6 ± 30.8	36.5 ± 19.1	0	0	0	0
ZDV300/3TC150	9.7 ± 13.9	34.5 ± 14.8	27.1 ± 43.1	(0, 143)	34 ± 29.5	(0, 52)
ZDV300/3TC150/NVP200	13 ± 19.9	20.5 ± 4.9	14.8 ± 33.6	(0, 93.6)	50 ± 0	(50,50)
3TC30/ZDV60/NVP50	15 ± 17	0	0	0	0	0
NVP 240 ml	33.7 ± 46.7	29 ± 41	5.9 ± 16.6	(0, 47.1)	0	0
D4T12/3TC60/NVP100	20.1 ± 25	27 ± 0.0	27.8 ± 83.5	(0, 290)	0	0
D4T6/3TC30/NVP50	16.8 ± 24.4	0	110.8 ± 193.8	(0, 400)	0	0
3TC30/ZDV60	14.8 ± 21.8	16.5 ± 14.8	25.4 ± 35.9	(0, 50.8)	45.7 ± 12.7	(36.7,54.7)
D4T 12/3TC60	15.6 ± 33.1	0	0	0	69.4 ± 33.3	(45.8,93)
D4T 6/3TC30	55.8 ± 45	0	^a^	^a^	0	0

^a^means the product was not ordered by the health facility in last requisition and reporting reviewed

*D4T = Stavudine; EFV = Efavernez; NVP = Nevirapine; TDF = Tenofovir; 3TC = Lamivudine; ZDV = Zidovudine*

### Supportive supervision

All of the health centers and 3/4^th^ of the hospitals were supervised by professionals from the Regional Health Bureau or FMoH during the past 6 months prior to the study. The study also showed that only 2 of the hospitals and a quarter of the health centers had received supportive supervision more than 3 months ago. All of the respondents said that their last supervision included review of stock cards and bin cards, physical stock count, storage condition, review of health commodity information management system (HCMIS) and EDT, PIS and dispensing register book, VCT tally and VCT daily register. They also discussed and facilitated removal of expired products from the stores.

### Storage condition of ARV medicines and test kits

The stores of 19 health centers and 4 hospitals were assessed during the study time. There were 2 health centers which didn’t have a bin card for ARV medicines and not included in the calculation of percentage of bin card updated. There were also 2 hospitals, which had updated bin card with 1 month and 2 months transaction; which were included in the calculation of availability of bin card for ARV medicines and bin card updated. But these two hospitals had no complete transaction for the past 6 months. Store manager of one of the hospital had a complete bin card of 9 ARV medicines, so only the bin card of the 9 products were used in the past 6 month stock status. The store manager of the second hospital didn’t record the past 14 months transaction neither on bin card nor on the HCMIS; and thus the hospital was excluded in the calculation of past six months stock status of ARV medicines. The stock status was then calculated with these limitations.

Overall, 14(73.7 %) of the health centers and 3(75 %) of the hospitals had stock out of one or more ARV medicines on the day of visit. All of the hospitals and health centers faced stock out of 1 or more ARV medicines in the past months prior to the study time. EFV600, NVP200, NVP240 and D4T6/3TC30/NVP50 were stocked out both at hospitals and health centers.

The average number of products which were out of stock on the day of visit was 1.6 and 2.0 in health centers and hospitals respectively and it went as high as 6 and 4 in health centers and hospitals respectively. Mean number of out of stock products in the past six months prior to the study was 5.1 and 6.5 in health centers and hospitals, respectively (Table 4). The most frequent out of stock item in the past 6 months prior to the study in the health facilities was TDF300/3TC300. The mean duration of stock out was longer for D4T6/3TC30 (55.8 ± 45 days, (0, 109)) and TDF300/3TC300 (45.9 ± 45.5 days, (0, 165)) in health centers while it was long for TDF300/3TC300 (42.5 ± 60.1 days, (0, 85)) and NVP200 (36.5 ± 19.1 days, (23, 50)) (Table 3).

**Table 4:** Proportion of ARVs managed and stocked out in in the health facilities on the day of visit and within 6 months prior to the study, Addis Ababa, 2013

	On day of visit				The past 6 months			
	Health centers		Hospitals		Health centers		Hospitals	
	Mean ± SD	(Min, Max)	Mean ± SD	(Min, Max)	Mean ± SD	(Min, Max)	Mean ± SD	(Min, Max)
No. of ARVs managed	12.2 ± 2	(8,14)	13.2 ± 3.8	(8,16)	12.2 ± 2	(8,14)	13.2 ± 3.8	(8,16)
No. of ARVs stocked out	1.6 ± 1.5	(0,6)	2 ± 1.6	(0,4)	5.1 ± 2.6	(1,11)	6.5 ± 2.1	(5,8)
% of ARVs stocked out	12.8 ± 11.3	(0,42.8)	17 ± 13.7	(0,30.8)	46.3 ± 23.3	(9.1,91.7)	56.3 ± 8.8	(50,62.5)

Decreasing the ordered quantity of ARV medicines and test kits by the supplier was mentioned as a main reason for the stock outs of these pharmaceuticals. In addition to this, respondents claimed that the transfer of patients from D4T to TDF/3TC based regimen was a major contributing factor for shortage of TDF300/3TC300.

According to majority of the store managers, stock movement was controlled using both bin card and HCMIS. Except 2 (10.5 %) of health centers; all of the health facilities used bin card on the day of visit. Besides, all health facilities used maximum-minimum stock control system to manage the stock of ARV medicines and as a result they were supposed to have maximum stock of 4 months, minimum stock of 2 months and an emergency stock of 15 days.

During the time of visit, average percentage of updated bin cards were 85.7 % in health centers and 96.9 % in hospitals. Percentage of bin card updated varied from facility to facility with a range of 22.2– 100 % in health centers and 87.5–100 % in hospitals. All bin cards were updated in 3(75 %) of the hospitals and 10(58.8 %) of health centers. While the remaining hospitals and health centers had one or more un-updated bin cards.

**Fig. 1:**
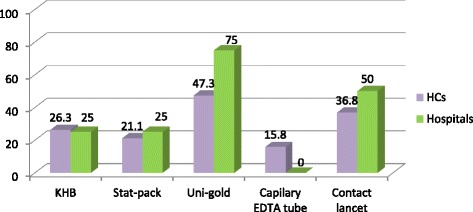
Proportion of health facilities stocked out of HIV test kits on the day of visit in the, Addis Ababa, 2013

Regarding the selected HIV test kits, (KHB, stat-pack, Uni-gold, blood lancet and EDTA capillary tube), at the time of the assessment only 10(52.6 %) of health centers and 2(50 %) of hospitals had a bin card. Regarding the stock status of test kits, 10(52.6 %) of the health centers reported stock-out of 1 to 3 test kits and all hospitals had stock out of 1 or 2 test kits. In a majority of the health centers and hospitals, Uni-Gold was out of stock on the day of visit more than other kits (Fig. 1). Average percentage of test kits which were out of stock on the day of visit was 29.5 % and 35 % in health centers and hospitals, respectively.

Except one hospital, all of the facilities had one central store for ARV medicines and test kits. The qualitative data obtained from observation checklist showed that appropriate arrangement of products with visible expiry dates and identification labels, first expired first out (FEFO) organization of product and accessibility of products for counting; cleanliness of the store, and thermometer usage were the major challenges identified in majority of the health facilities. Lack of ventilation, inadequate light of the store room and inadequacy of storage space were observed in a majority of the health centers and few hospitals. The stores in a majority of the health centers were highly overfilled and products were kept in a direct floor without a pallet. In addition, there was sign of rodents and insects in the majority of health centers and few of the hospitals.

It was also observed that, utilization of expiry tracking chart was very minimal both in the health centers and hospitals. Expired medicines and test kits were found on the shelves of some of the health centers and one hospital. On the contrary, majority of the health centers and hospitals were able to maintain the outer cartoon of the product in a good condition and they were able to separate the expired and damaged product from usable product either in the store room or in separate store. Moreover, the observation revealed that cartoons and products were protected from direct sunlight; stores were locked and the keys were maintained with the store manager, roofs were maintained in good condition to avoid sunlight and water penetration.

### Logistic management information system

For majority of the health centers and hospitals, EDT was the main tool to record patient information even though there was risk of power interruption and risk of losing the patient data. EDT contains all the medicines currently dispensed at facilities unlike HCMIS. Since there is large number of HIV/AIDS patients, this computerized software eases the work of the health professionals and the data clerks at the dispensary. However, the professionals had reported drawbacks of the software; not easy to manipulate and get patient data by regimen type. So when the need arises, they relied on the data obtained from the clinic which had also some shortcomings.

Problem of data quality was also reported by majority of the health facilities. Only one health center was able to correctly report the ending balance as the stock on hand found in the central store room as well as in all other dispensing units, while the rest of the health facilities wrongly reported the ending balance as stock on hand kept in the store room only. Besides, deliberate manipulation of recording was reported by one store manager as there were cases where the supplier sent excess ARV medicines and thus the manager targeted to decrease the ordered ARV medicines. Hence, to prevent and minimize the fluctuations made by the supplier, the store manager of the health centers increased or decreased the balance to be reported accordingly.

## Discussion

Although information about patients taking ARVs medicine by regimen data is crucial for the medicine supply chain management, it was only 16 (80 %) of the health centers and one hospital that were able to know the patient medication record data and reported appropriately. The main problem was related to inefficiency of the EDT and as a result some of the health facilities were using the ART clinic data as an alternative. Nevertheless, the report from the ART clinic lacked patient data on TDF/3TC/EFV and TDF/3TC/NVP; where these two medicines accounted near to quarter or more of the patients in the health facilities and other second line ARV medicines. Thus, this lack of adequate and accurate data might affect quantification and procurement planning for ARV medicines. Similarly, a study done by Al1ers et al. showed that, data on ART services and ARV medicine supply were limited and, when available, are often unreliable or insufficient to be used for quantifying ARV medicine requirements [7]. Another literature stressed the importance of information in medicine supply chain management to ensure that there are no interruptions in treatment and tests [5].

Adherence is an important issue in any antibiotic therapy but it is of a special concern when it comes to medicines like ARVs. HIV patients should be monitored and followed with much of concern. In this regard, the use of patient tracking chart is vital tool to monitor the adherence and thus recommended by the standard operating procedure for management of ART medicines in health facilities [8]. The present study, however, showed that only half of the health centers and hospitals had been using patient tracking chart. In those facilities which didn’t use patient tracking chart, medicines were simply dispensed to patients who came to their dispensary but they didn’t know how much of their patients were missed and treatment adherence is left to the patients. This practice could contribute to default of more patients, probably emergence of medicine resistance and loss of lives.

Our study also documented that in the studied health facilities, ART dispensers were dispensing doses that range from 15 days to 3 months depending on the patient’s condition. Frequent stock-out of medicines and regimen change from D4T to TDF/3TC which is associated with shortage of TDF/3TC, however forced them to dispense medicines even for a single day. The percentage difference between quantities ordered and received was high for TDF/3TC both in health centers and hospitals. Such high percentage difference between ordered and received quantity was an indicative of interrupted supply chain. This kind of practice therefore might exhaust the patients and contribute for default. The present study result mirrors to a study in Mali, where forecasting became difficult due to shifting of patients to alternative first line and second line ART regimens [9].

Accurate quantification of HIV/AIDS related commodities is a complex process confined with multifaceted factors [17]. The present study revealed that even though both health centers and hospitals control their inventory using maximum-minimum stock inventory control system, frequent emergency order of ARV medicines was reported in the six months prior to the study. A majority of the health centers had one or more emergency orders while all of hospitals had emergence orders of ARV medicines more than three times. This might be associated with the relatively higher number of patients in hospitals than health centers.

This study also showed that only half of the hospitals and a quarter of the health centers had received recent supportive supervision more than 3 months ago. So, lack of planned and timely supportive supervision may also become a challenge in ensuring adequate supplies. A study done in Lesotho similarly revealed that there were challenges in the medicine supply management system, which were mainly due to the lack of supervisory site visits, leading to facilities over-stocking or under-stocking of certain items [18].

Failure to observe expiry dates of medicines might lead to the loss of a significant amount of resources, especially in resources limited countries. This type of loss is not acceptable to pharmaceuticals such as ARV medicines, which are very expensive [8]. Contrary to this fact, the present study showed that utilization of expiry tracking chart was minimal in health facilities and thus on the day of visit, expired medicines and test kits were found on the shelves of the health facilities. Similar to this finding, a study done in Lesotho showed that, ARV medicines were expired on the shelves in some facilities where inventory was poorly managed [18].

The present assessment also indicated that more than three fourth of the hospitals and health centers had stock-out of one or more ARV medicines on the day of visit and during the past 6 months preceding the survey date. TDF300/3TC300 was most frequent stock out item in the past 6 months both in hospitals and health centers. Mean number of stock out products in the past 6 month was 5.1 and 6.5 in health centers and hospitals, respectively. The percentage of products which were out of stock was 12.8 % and 17 % in health centers and hospitals, respectively. Regarding the test kits, all of the hospitals and 2 of the health centers reported stock out of one or more test kits on the day of visit. Different from this study, a study done in Oromia National Regional State showed that availability of first line ARV medicines was 100 % in health centers and 95 % in hospitals [19]. Another study done in Ethiopia also showed that stock outs for ARVs had been non-existent or minimal [14]. An assessment done in Sierra Leon similarly showed that there were stock-outs of EFV and second line medicines in ART providing facilities [7]. Concur to our finding, a study done in Uganda showed that, ARV shortages affected all ART providing facilities with considerable fluctuations. ARVs were available at 83 % and diagnostic kits at 70 % of the health facilities surveyed [20]. Similarly, Jonathan et al., showed that interrupted supplies and stock outs were the major challenges in the supply chain of ARV medicines in Africa [21].

Interruption of the supply of these medicines put individual patient at risk of disease progression and death, in medicine resistance development, hampers progress towards universal access, and diminishes the credibility of ART programs in the eyes of patients, community and healthcare providers and inadvertently putting the public health in danger [10]. So to prevent this kind of interruption, there has to be efficient supply chain. Effective medicine supply chain management and inventory control would help to avoid or at least minimize stock-outs, losses due to unnecessary expiry, theft and ensure that the desired pharmaceutical products are available at all times in adequate quantity [8].

Except 2 (10.5 %) of health centers; all health centers and hospitals used bin card for ARV medicines on the day of visit. Availability and practice of updating of the bin cards was relatively better in hospitals than health centers. Unlike ARV medicines, only 10(52.6 %) of health centers and 2(50 %) of hospitals had a bin card for the selected test kit on the day of visit. Consistent to this findings, a study done in Sierra Leone reported that the stock keeping practice in ART providing health facilities was not good where no bin cards were available for any of the ARV medicines or HIV test kits at any of the health facilities visited [22]. Contrary to the present findings, however, a 2009 evaluation done in Ethiopia indicated that the inventory control in all surveyed ART sites and use of bin cards and stock cards was found to be adequate [14].

To provide clients with high quality products and ensure efficient handling and use of products, each facility must have safe, protected and organized storage areas. The present assessment however, revealed that the storage facilities both in hospitals and health centers were far from adequate. The store premises and storage conditions were better in hospitals compared to health centers. Similarly, a study done in Sera Leon also stated that the storage condition observed in district and primary healthcare units was not generally in a good condition where expired medicines and kits were stored together with the usable commodities causing shortage of space in the health facilities [22]. An evaluation done in Ethiopia similarly showed that there were inadequate storage facilities, management, capacity, and temperature monitoring, especially for the cold chain [14].

The health facilities in Addis Ababa had both computerized and paper based LMIS. However, a majority of the health facilities had problems with the use of the automated LMIS. According to them, some features of the software were not easy to manipulate and fix the problem. As a result, relying on the computerized LMIS leave majority of the health facilities with incomplete and inaccurate data in both dispensary and store areas. A study suggested that computerized LMIS can greatly facilitate the work of supply chain managers and hence there is urgency to introduce user-friendly tools and software to support the management of logistics information system in the health facilities [23].

## Conclusions

ART and VCT services started in Ethiopia over a decade ago. However, data on patient medication record and stock status of ARV medicines and Test kits is still inadequate. The present study demonstrated that a majority of the facilities in Addis Ababa didn’t have patient tracking chart and consequently treatment adherence seemed to be left to the patients themselves. The reporting and receiving system of ART medicines were relatively more organized compared to HIV test kits in the studied health facilities. Though the stock status of ART medicines were controlled relatively in better way than HIV test kits, shortages of both commodities were common in both health centers and hospitals. Of all commodities; TDF/3TC, KHB and Uni-gold were the major out of stock items in the supply chain. For example, more than half of the health facilities were not providing VCT service on the day of our visit mainly due to lack of KHB. The storage condition of these commodities was not good but it was relatively better in hospitals than health centers. On the other hand, the stores and ART pharmacy sections of all health facilities had computerized LMIS. However, majority of the professionals who were supposed to run the system claimed that they were unable to manipulate and operate the software efficiently. Hence, introducing user-friendly tools and software is essential to support the management of logistics information system in the health facilities.

## Abbreviations

AA: Addis Ababa
AIDS: acquired immune deficiency syndrome
ART: antiretroviral therapy
ARVs: antiretroviral
D4T: stavudine
EDT: electronic dispensing tools
EDTA: ethylenediaminetetraacetica acid
EFV: efavernez
FEFO: first expired first out
FMOH: federal ministry of health
HCMIS: health commodities management information system
HIV: human immunodeficiency virus
IPLS: integrated pharmaceuticals logistic system
KHB: HIV (1 + 2) antibody colloidal gold
LIAT: logistic indicator assessment tools
LMICs: low and middle income countries
LMIS: logistic management information system
NVP: nevirapine
PFSA: pharmaceutical fund and supply agency
PIS: patient information sheet
PMTCT: prevention of mother to child transmission
RRF: report and requisition format
TDF: tenofovir
3TC: lamivudine
VCT: voluntary counseling and testing
WHO: World Health Organization
